# Evaluation of IL-1β levels in epilepsy and traumatic brain injury in dogs

**DOI:** 10.1186/s12868-019-0509-5

**Published:** 2019-06-17

**Authors:** Draginja Kostic, Regina Carlson, Diana Henke, Karl Rohn, Andrea Tipold

**Affiliations:** 1 Department of Small Animal Medicine and Surgery, University of Veterinary Medicine Hannover, Foundation, Buenteweg 9, 30559 Hannover, Germany; 2 Present Address: Animal Clinic am Hasenberg, Stuttgart, Germany; 3 Institute of Biometry, Epidemiology, and Information Processing, University of Veterinary Medicine, Hannover, Germany; 4 Centre for Systems Neuroscience, Hannover, Germany

**Keywords:** Interleukin-1 beta, Epilepsy, Traumatic brain injury, Cerebrospinal fluid, Serum, Canine

## Abstract

**Background:**

Epilepsy is a common neurological disease in dogs affecting approximately 0.6–0.75% of the canine population. There is much evidence of neuroinflammation presence in epilepsy, creating new possibilities for the treatment of the disease. An increased expression of interleukin-1 beta (IL-1β) was reported in epileptogenic foci. We hypothesized that there is an elevation of IL-1β in serum and CSF of dogs with epilepsy, as well as in serum of dogs with TBI, reflecting involvement of this cytokine in pathophysiology of naturally occurring canine epilepsy in a clinical setting.

**Results:**

IL-1β levels were evaluated in CSF and serum of six healthy and 51 dogs with epilepsy (structural and idiopathic). In 16 dogs with TBI, only serum was tested. IL-1β concentrations in CSF were not detectable. Serum values were not elevated in dogs with TBI in comparison to healthy controls (*p* > 0.05). However, dogs with epilepsy had increased levels of IL-1β in serum (*p* = 0.003) regardless of the underlying cause of the disease (*p* = 0.0045). There was no significant relationship between the variables and IL-1β levels. Statistically noticeable (*p* = 0.0630) was that approximately 10% of dog with epilepsy (R^2^ = 0.105) had increased seizure frequency and IL-1β elevation.

**Conclusion:**

Increased IL-1β levels were detected in the peripheral blood in dogs with idiopathic and structural epilepsy leading to the assumption that there is an involvement of inflammation in pathophysiology of epilepsy which should be considered in the search for new therapeutic strategies for this disease. However, to better understand the pathogenic role of this cytokine in epilepsy, further evaluation of IL-1β in brain tissue is desired.

## Background

Cytokines are important signaling molecules [1] involved in the immunity, inflammation and hematopoiesis, but also in the functional alteration of cells in the central nervous system (CNS) [2]. Cytokines are well known to strongly influence signaling processes in the CNS during injury, inflammation or disease [3, 4]. They can be pro- or anti-inflammatory [5].

Interleukin-1 beta (IL-1β) belongs to IL-1 family of pro-inflammatory cytokines and plays an essential role in injury and inflammation [6]. In the CNS it is mainly produced by activated microglia [7], but also neurons [8], astrocytes [9] and oligodendrocytes [10]. There is ever-growing knowledge of this interleukin’s activity in healthy as well as inflamed brain parenchyma [11]. In the healthy brain, IL-1β levels are low, but detectable [12] suggesting a certain function in the CNS physiology such as sleep [13, 14], learning and memory [15], as well as neuromodulation on different levels of cells communication in the CNS [11, 16, 17]. In the CNS diseases, involvement of IL-1β is described in neurodegeneration [18, 19], depression [20], neuro-trauma [21] and epilepsy [22, 23]. In chronic and acute inflammatory processes in the CNS, it plays both, a beneficial and a harmful role [11] and therefore could represent a target for drug development [24, 25].

Epilepsy is a common neurological disease in dogs affecting approximately 0.6–0.75% of the canine population [26]. Current treatment options for the disease are limited and aim for a reduction of seizure frequency not influencing the pathophysiology [27]. There is much evidence of the presence of neuroinflammation in epilepsy [23]. The potential involvement of IL-1β in inflammatory reactions in epilepsy has attracted considerable attention and despite equivocal reports on its implication in seizures [28], it presents a possibility for characterizing new treatment options [29]. For instance, blocking of the IL-1β signaling is reported to prevent status epilepticus in epileptic patients [30].

Traumatic brain injury (TBI) is considered a global health problem and is known to have detrimental consequences in human [31, 32] and veterinary medicine [33], such as cognitive impairment [34] or the development of post-traumatic epilepsy. TBI can cause inflammatory reactions in the brain [35] and subsequently lead to epileptogenesis [23, 36]. It has been suggested that increased IL-1β levels during inflammation after TBI have predictive value for development of post-traumatic epilepsy (PTE) [37].

In veterinary medicine, IL-1β concentration in peripheral blood has been described as a possible marker for early stages of inflammation in dogs [38]. Increased expression of IL-1β in the CNS has been reported in dogs with acute spinal cord injury (SCI) in choroid plexus [39], in the brain lesions of animals with canine distemper virus infection [40] and the brain parenchyma of dogs with TBI [41]. In dogs with degenerative myelopathy, a decrease of IL-1β in plasma was recorded [42].

Based on the reported IL-1ß involvement in the epilepsy models and the potential predictive value this cytokine could have for PTE, this study focus was to determine the possible role of IL-1β in canine epilepsy and TBI. Therefore, concentration of IL-1β in the peripheral blood of dogs with traumatic brain injury and epilepsy was investigated, as well as its presence in cerebrospinal fluid (CSF) of dogs with epilepsy using an Enzyme-Linked Immunosorbent Assay (ELISA). We hypothesized that there is an elevation of IL-1β in serum of dogs with TBI as well as in serum and CSF of dogs with epilepsy, reflecting involvement of this cytokine in the pathophysiology of naturally occurring canine epilepsy in a clinical setting.

## Results

IL-1β concentration was evaluated in a total of 73 dogs included in the study. In all CSF (n = 57) samples IL-1β could not be detected using the described ELISA.

In the healthy dogs (n = 6), two serum samples had detectable IL-1β levels. The mean concentration, SD and the range of IL-1β in these control samples were 14.8 ± 23.4 (0.0–58.0) pg/mL (Table 1).

**Table 1:** Mean values of IL-1β in serum (pg/mL)

Diagnosis	Healthy (n = 6)	TBI (n = 16)	Idiopathic epilepsy (n = 30)	Structural epilepsy	
				Inflammation (n = 9)	Tumor (n = 12)
Mean values ± SD (range)	14.8 ± 23.4 (0.0–58.0)	92.6 ± 75.1 (12.9–248.0)	118.6 ± 81.4 (0.0–312.0)	134.9 ± 79.2 (48.0–237.0)	154.2 ± 93.7 (37.5–308)

*TBI* traumatic brain injury, *SD* standard deviation

In all dogs with TBI (n = 16) only serum samples were evaluated and in all samples IL-1β was measurable. The mean value, SD and the range of IL-1β concentration in serum of TBI dogs were 92.6 ± 75.1 (12.9–248.0) pg/mL (Table 1). The range of MGCS score was from 3 to 18, with the mean score of 13 (SD = 4.01). However, after applying Kruskal–Wallis test to compare levels of IL-1β in serum of healthy dogs and TBI dog, it was concluded that there was no significant difference between these two groups (*p* > 0.5) despite the single elevated values.

The animals with epilepsy were divided in the idiopathic and structural epilepsy group. In all dogs (n = 51), IL-1β was measurable, except for two with idiopathic epilepsy. Animals with idiopathic epilepsy had the mean values, SD and the range of serum IL-1β of 118.6 ± 81.4 (0.0–312.0) pg/mL (Table 1). IL-1β in serum of dogs with inflammatory brain disease and brain tumor showed the mean values, SD and the range of 134.9 ± 79.2 (48.0–237.0) pg/mL and 154.2 ± 93.7 (37.5–308) pg/mL, respectively (Table 1).

**Fig. 1:**
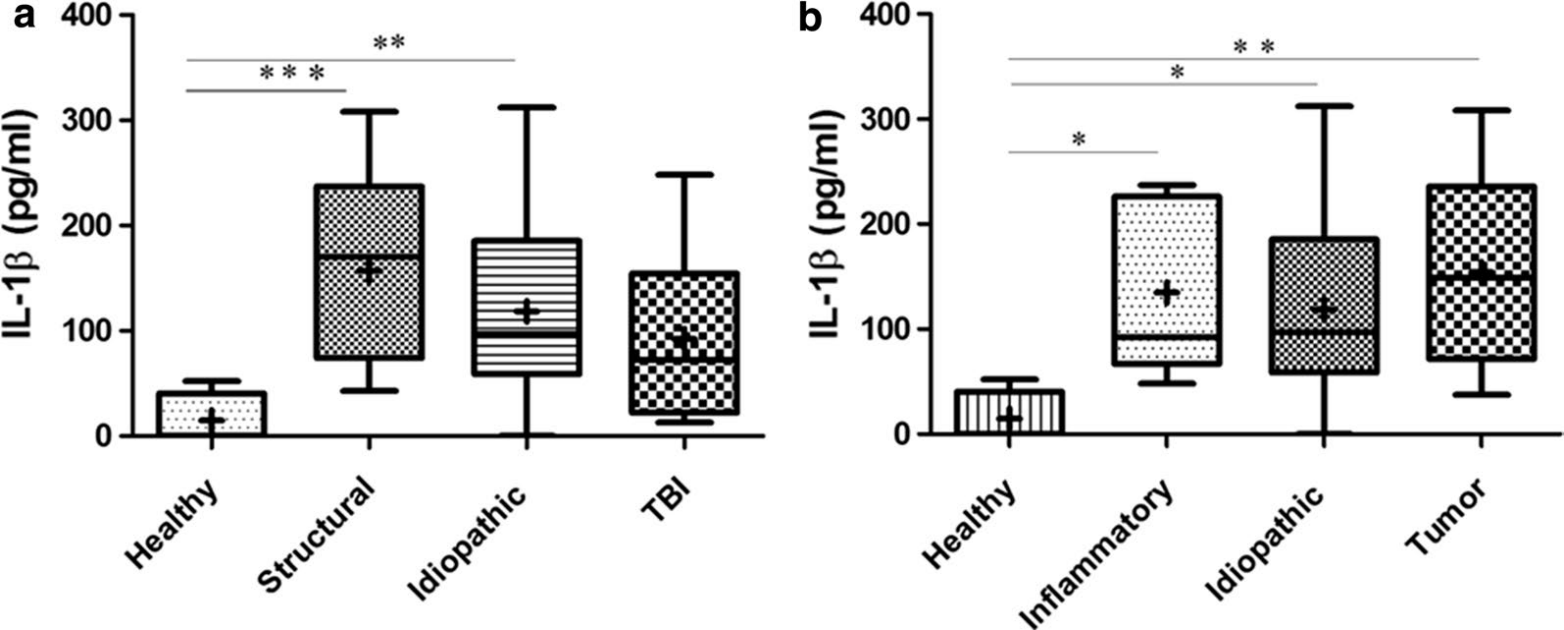
**a** Levels of interleukin-1 beta (IL-1β) in serum of healthy dogs compared to dogs with idiopathic and structural epilepsy and traumatic brain injury (TBI). **b** Level of interleukin-1 beta (IL-1β) in serum of healthy dogs compared to dogs with idiopathic epilepsy and dogs with inflammation and tumor in the brain; boxes contain values from the first to third quartile of serum IL-1β levels pg/ml. Lines inside of the box indicate median values, and (+) represents mean values; Lines outside of the box indicate maximum (above the box) and minimum (beneath the box) observation; The asterisk mark statistically significant difference after Bonferroni correction (*p < 0.05; **p < 0.01; ***p < 0.001)

In order to compare levels of IL-1β in serum samples of epileptic to those of healthy dogs, Kruskal–Wallis test for unequal sample size was performed and showed that both idiopathic as well as structural epilepsy dogs had higher levels of IL-1β in serum than healthy dogs (*p* = 0.003; Fig. 1a). However, there was no statistical difference between the levels of IL-1β in structural and idiopathic epilepsy group (*p* > 0.05; Fig. 1a). The same test was used to compare IL-1β serum levels in healthy dogs to those of the dogs with idiopathic epilepsy, inflammatory brain disease and brain tumors, and each group had higher IL-1β levels than controls regardless of the underlying cause of the seizures (*p* = 0.0045; Fig. 1b.) When compared to each other, there was no statistically significant difference between dogs with idiopathic epilepsy, inflammatory brain disease or neoplasia regarding their levels of IL-1β in serum (*p* > 0.05; Fig. 1b).

Linear regression analysis of IL-1β levels and the time point between sample collection and the last seizure event (mean = 4.3 days, SD = 4.7) resulted in the R^2^ = 0.0014 and with *p* = 0.8963 was statistically not significant (Table 2). The same was concluded for the linear regression analysis of relationship between levels of IL-1β in epileptic dogs and the duration of the disease (mean = 95.2 day, SD = 78.5, R^2^ = 0.0097, *p* = 0.6112). Interestingly, the linear regression analysis of L-1β levels and seizure frequency showed statistically noticeable, but not significant relationship, with R^2^ = 0.105 and *p* = 0.0630 (mean = 3.8 seizures per month, SD = 3.4) (Table 2). In dogs with different type of seizures, respectively different seizure severity, no statistically significant difference (*p* = 0.7164) was calculated, using the one-way ANOVA test regarding the levels of IL-1β (in dogs with single seizures: mean = 95.83 pg/mL, SD = 94.38; cluster: mean = 97.17 pg/mL, SD = 61.81; status epilepticus: mean = 123.2 pg/mL, SD = 87.94).

**Table 2:** Analysis of IL-1β in serum of epileptic dogs and different variables

	R^2^	*p* value	Mean (SD)
Time point between sample collection and last seizure event (n = 14)	0.0014	0.8963	4.3 (4.7)
Duration of the disease (n = 29)	0.0097	0.6112	95.2 (78.5)
Seizure frequency (n = 32)	0.105	0.0630	3.8 (3.4)
Type of seizure	n/a	0.7164	
Single (n = 12)			95.8 (94.38)
Cluster seizures (n = 9)			97.17 (61.81)
Status epilepticus (n = 5)			123.2 (87.94)

R^2^, coefficient of determination

## Discussion

In the current study IL-1β was measured in serum and CSF of dogs with epilepsy, which is to the authors’ knowledge the first canine-based study. IL-1β levels should be evaluated to prove the occurrence of an inflammatory reaction in canine epilepsy. In addition, the concentration of the IL-1β was also measured in serum of TBI dogs, as these animals tend to develop post traumatic epilepsy [43].

In all dogs with epilepsy, as well as in healthy dogs, IL-1β was not measurable in CSF using the described ELISA. Based on the origin of metabolites in the CSF, an association between the occurrence of IL-1β in CSF and the brain tissue would have been highly plausible and could tell more about the role of IL-1β in epilepsy [28]. However, in the current study, similar to the report in human, the low sensitivity of the ELISA tests, the time between sample collection and the last seizure event and different causes of the disease most probably prevented the detection of the cytokine in the CSF samples [28]. The time interval between the last seizure event and sample collection (mean value = 4.3 days) seems not to have influenced the values in canine serum, which is similar to the recent study in human patients [44]. Regarding veterinary research of other CNS diseases, another attempt to asses IL-1β in CSF of dogs with degenerative myelopathy failed since the values were below the detection limit of the ELISA [42].

Epileptogenesis is a subject of a great scientific interest. Better understanding of this process offers numerous possibilities of revealing the disease cause and for new treatment approaches. After severe TBI, a high percentage of human and canine patients develop PTE (20% resp. 14.3%) [43, 45]. During the process of disease development, an increase of IL-1β occurring in the injured brain tissue was described [36, 46]. Thus, we were interested to measure IL-1β concentration in TBI in the peripheral blood and tried to confirm that a spillover from the CNS occurs in the first 2 days after injury and can be evaluated in a clinical setting. However, there was no statistical difference between IL-1β serum values of healthy dogs and dogs with TBI, although IL-1β was measurable in each sample in comparison to the controls. Also, in single cases, very high levels of IL-1β were detected. A few of these cases had very low MGCS score which is predictive of the not favorable outcome (dead or alive) [47]. Nevertheless, based on our results, we could only speculate as to whether or not these high levels of the IL-1β in serum of the single cases could point to potential development of PTE. In human medicine, intracranial levels of IL-1β are significantly higher than in plasma in TBI patients and the production of cytokines in the CNS seems to be highly compartmentalized [48]. This could explain low levels of IL-1β in our serum samples, despite its increased production in the brain reported by Yu at al. [41]. Relatively low number of TBI dogs included in our study as well as the heterogeneous population regarding severity of injury could have also affected our results. We are likewise aware of our study design limits concerning the use of one breed for the reference values. However, Prachar et al. [38] also reported only few IL-1β positive serum samples in the heterogenous healthy canine control group.

Pro-inflammatory cytokines and IL-1β are potentially involved in the pathophysiology of epilepsy [29]. Experimental research associated the IL-1β production in epileptogenic brain areas with acute and subsequently, chronic neuroinflammation in epilepsy [23]. With premise that it mirrors the inflammation in epilepsy, we have evaluated levels of IL-1β in CSF and serum of dogs with idiopathic and structural epilepsy. Although the cytokine was not detectable in the CSF, in the serum samples of dogs with epilepsy significantly elevated levels were detected when compared to healthy controls. Interestingly, there was no difference between dogs with idiopathic and structural epilepsy. This remarkable result suggests that regardless of the cause of epilepsy, IL-1β is elevated in blood. Also, the increased IL-1β in the blood of dogs with epilepsy, confirms the involvement of inflammation in the pathophysiology of the disease. The neuroinflammation in epilepsy presents inflammatory response of the brain tissue to a neurogenic activity, i.e. seizures [49]. Such an acute response includes the release of IL-1β and other cytokines which in turn can help the brain to maintain homeostasis or harmfully perpetuate and spread chronic inflammation, neuroexcitability and weaken the blood–brain barrier [50]. The leakage of the BBB that occurs, could lead to the increase of the IL-1β in blood [51], which explains the cytokine detected in our dogs with epilepsy. The source of IL-1β in the peripheral blood could also represent a mixture between the central and the peripheral response of the immune system to epilepsy [37], by the activation of microglia as well as peripheral monocytes.

Increased levels of IL-1β in serum were detected and especially single cases displayed high values, in both structural and idiopathic epilepsy. There have been reports in human medicine of presumed idiopathic epilepsy cases, which in fact proved to be immune-mediated [52]. This could explain the high value of IL-1β in the single cases of idiopathic epilepsy. Similar results have been found evaluating IL-17 in serum and CSF of dogs with idiopathic epilepsy [53]. Nonetheless, further association with different variables in epilepsy was needed to better explain the role of the cytokine. Considering that the seizures present the common denominator for the three evaluated groups of epileptic animals, their association with IL-1β levels was evaluated through the following variables: time point between sample collection and the last seizure event, duration of the disease, seizure frequency, and type of seizure resp. seizure severity. However, no significant relationship between the variables and IL-1β levels could be calculated. Nevertheless, it was statistically noticeable (*p* = 0.0630) that approximately 10% of the dogs with epilepsy (R^2^ = 0.105) had increased seizure frequency and IL-1β elevation. Similar results were described in human medicine and the dependency between the seizure frequency and IL-1β production occurred [44, 54]. Regardless, there are still controversial reports on the exact role and the mechanism of the influence of IL-1β on seizures in epilepsy [28, 51]. The fact that there was no relationship between level of IL-1β and the time point of the sample collection or the duration of the disease could be explained by potential constant chronic inflammation without IL-1β level fluctuations. In addition, Gao et al. suggested that no interictal and postictal alteration of the cytokine’s level in peripheral blood in epilepsy occurs [44]. No differences between seizure types regarding IL-1β levels could be proven, although those differences might be better evaluated in the first hour after the event [55].

## Conclusion

In this study, we have detected increased IL-1β in serum of dogs with epilepsy regardless of the cause. There is a constant challenge in finding new treatment options for epilepsy, considering multiple etiology and interindividual differences [56]. This fact could be used for further therapy attempts. However, we could not detect IL-1β in CSF or make a connection between serum levels and seizures. Also, there was no change in serum level of IL-1β in dogs with TBI. Bearing that in mind, we suggest direct measurement of the IL-1β in brain parenchyma of epileptic dogs, to better understand its role in seizures and epilepsy. Also, among the presumed idiopathic epilepsy cases, single dogs with very high levels of IL-1β, could in fact have immune-mediated epilepsy which needs more in-depth research.

## Methods

### Animals inclusion and sample collection

The study included paired serum and CSF samples of six healthy beagles, 51 epilepsy and 16 TBI dogs presented between 2013 and 2016 at the Small Animal Clinic of the University of Veterinary Medicine Hannover, Germany. Six TBI samples were kindly provided by Dr. Diana Henke, from Vetsuisse Faculty, University in Bern. Samples from the diseased dogs were collected after owner’s written consent. The study design followed ethical guidelines of the University and procedures on healthy dogs were approved by the authorities of Lower Saxony (animal experiment number 33.9-42502-05-14A453).

Dogs were selected based on history and available diagnostic data. All animals in the study underwent clinical and neurological examination, complete blood testing and different imaging studies. Depending on the presumed or confirmed diagnosis, the dogs were divided in the following groups: idiopathic epilepsy, structural epilepsy, TBI, healthy (Table 3).

**Table 3:** Groups of the dogs according to diagnosis

Diagnosis	Findings and number of dogs
Idiopathic epilepsy	Seizures and normal interictal general, neurological, MRI and CSF examinations; n = 30
Structural epilepsy	Seizures and presumed inflammatory brain disease; n = 9
	Neoplastic brain diseases; n = 12
TBI	History of head injury, clinical, neurological and imaging examinations; n = 16
Healthy	Normal physical and neurological examination; n = 6

*MRI* magnetic resonance imaging, *CSF* cerebrospinal fluid, *TBI* traumatic brain injury

In the study were included 22 female, 10 neutered female, 35 male and 6 neutered male dogs. Age of the dogs was in range between 6 months and 14 years and various breeds were encompassed with ten mix breeds, nine beagles, three Border Collies, three Golden Retrievers, and miscellaneous like bull terrier, dachshund, French bulldog, Jack Russel terrier, Labrador Retriever, Siberian husky, Irish setter, German shepherd, Australian shepherd.

Control group consisted of healthy, clinic owned beagles with normal clinical and neurological examination, normal blood values as well as normal CSF analysis.

Dogs with epilepsy were classified with presumed or confirmed structural or idiopathic epilepsy according to recommendations for standardized diagnosis by the International Veterinary Epilepsy Task Force [57]. Clinical data about seizures in epileptic dogs such as type of seizure, duration and frequency were collected upon owner’s and/or neurologist’s observations (hospitalized cases).

In order to diagnose the idiopathic epilepsy (n = 30) in dogs tier 2 level of confidence was applied [57]: animals had a history of two or more unprovoked epileptic seizures, age at onset of the disease was between 6 months and 6 years, interictal general and neurological examination were unremarkable, results of blood tests, urine analysis, ultrasound and radiographic examination, as well as MRI and CSF analysis were normal. Clinical data such as duration of the disease, seizure frequency, seizure severity (single generalized seizures, cluster seizures or status epilepticus), time point between sample collection and last seizure event were recorded.

The group of dogs with the structural epilepsy consisted of animals with inflammatory CNS disease (eight dogs with meningoencephalitis of unknown origin (MUO) and one with bacterial encephalitis) and neoplasm of brain tissue presumably causing the observed seizures.

All animals diagnosed with structural epilepsy (n = 21) underwent physical and neurological examination, blood work as well as MRI and CSF tests. The presumed diagnosis ensued following recommendations [58] to diagnose either brain tumors [59] or brain inflammation [60] in dogs.

In the study, 16 dogs with TBI were included. Traumatic brain injury was diagnosed when dog’s history indicated recent (3–48 h) head injury, thorough physical and neurological examination and corresponding imaging findings [61]. Level of consciousness, motor and brainstem function of each dog with TBI was graded using modified Glasgow Coma Scale (MGCS) [62]. The MGCS is a clinical coma scale for dogs. The least severe cases of TBI had the highest MGCS score (MGCS = 18) and the most severe cases had an assigned MGCS score of “3”.

In all epileptic dogs and healthy beagles, CSF was obtained via suboccipital puncture in general anesthesia. Saphenous and cephalic vein blood was collected, centrifuged at 14,000 rpm for 2 min and serum was separated. In dogs with TBI, only serum was tested. In cases with head injury a CSF tap could lead to deterioration of clinical signs and such procedures are therefore contraindicated [61]. Blood sampling in TBI dogs occurred 3–48 h after the injury and referral to the clinics. All samples were immediately aliquoted, frozen and stored at − 20 °C until measurement.

### IL-1β determination

IL-1β was measured in the paired CSF and serum samples of epileptic and healthy dogs, as well as in the serum of animals with TBI.

Concentration of IL-1β was evaluated using a canine specific ELISA test (Kit No. SEA563Ca; Cloud-Clone Corp, Houston TX, USA). The sandwich type ELISA was performed according to the manufacturer’s instruction manual. Briefly, in the wells pre-coated with antibody specific to IL-1β, 100 µl of sample was added, followed by biotin-conjugated antibody, specific to IL-β. Next, Avidin-conjugated to Horseradish Peroxidase is added and incubated. When 3,3′,5,5′-Tetramethylbenzidine (TMB) substrate is added, only wells that contained biotin-conjugated antibody, IL-1β and the enzyme conjugated avidin had changed color. After adding sulphuric acid, the color change of the final product was measured using plate reader (Synergy 2 multi-mode reader, BioTek, Vermont, USA). The measured optical density was then compared to the standard curve values and expressed in pg/mL. Detection range of the test was 7.8–500 pg/mL. Minimum detectable dose was 3.1 pg/mL.

### Statistical analysis

All statistical analyses were performed using statistics program package SAS^®^, version 9.2 (SAS Institute, Cary, NC, USA). IL-1β data did not follow a normal distribution. Therefore, nonparametric statistical tests were used and differences between all the groups were evaluated using Kruskal–Wallis test and Bonferroni’s post hoc correction. First, all groups of the animals were compared to healthy dogs and to each other. Afterwards all epileptic dogs together were compared to TBI and healthy group. Mean values with standard deviation were calculated for each evaluated group. The graphical presentation of the results was done using GraphPad Prizm 6 (GraphPad Software, Inc., La Jolla, USA).

To associate the IL-1β concentrations in serum to the important parameters in epilepsy, linear regression analysis was performed for the following variables: duration of the disease, seizure frequency, time point between sample collection and the last seizure event. The relationship was described with the coefficient of determination, R-squared (R^2^). The levels of IL-1β were compared between groups with different seizure types respectively seizure severity (single, cluster seizures or status epilepticus) applying one-way ANOVA test.

## Data Availability

The datasets generated and/or analyzed during the current study are available in the G-NODE repository https://web.gin.g-node.org/Draginja/123.

## Abbreviations

ELISA: Enzyme-Linked Immunosorbent Assay
IL-1β: interleukin-1 beta
CNS: central nervous system
CSF: cerebrospinal fluid
PTE: post-traumatic epilepsy
SCI: spinal cord injury
TBI: traumatic brain injury
TMB: 3,3ʹ,5,5ʹ-tetramethylbenzidine
MGCS: modified Glasgow Coma Scale
MRI: magnetic resonance imaging
MUO: meningoencephalitis of unknown origin
